# Photo-Responsive Micelles with Controllable and Co-Release of Carbon Monoxide, Formaldehyde and Doxorubicin

**DOI:** 10.3390/polym14122416

**Published:** 2022-06-14

**Authors:** Bin Zheng, Lulu Yu, Huaze Dong, Jinmiao Zhu, Liang Yang, Xinsong Yuan

**Affiliations:** 1 School of Chemistry and Chemical Engineering, Hefei Normal University, Hefei 230061, China; dapdong@163.com (H.D.); jmzhu@ahu.edu.cn (J.Z.); yangliang@hfnu.edu.cn (L.Y.); yuanxs@zju.edu.cn (X.Y.); 2 Department of Polymer Science and Engineering, University of Science and Technology of China, Hefei 230026, China; yu123456@mail.ustc.edu.cn

**Keywords:** photo-responsive micelles, carbon monoxide, formaldehyde, doxorubicin

## Abstract

Endogenous gases have attracted much attention due to their potent applications in disease therapies. The combined therapy, including gaseous molecules and other medicines that can create synergistic effects, is a new way for future treatment. However, due to the gaseous state, gas utilization in medical service is still limited. To pave the way for future usage, in this work, an amphiphilic block copolymer containing nitrobenzyl ether, 3-hydroxyflavone (3-HF) derivatives and ether linker was constructed. The nitrobenzyl ether group endows the polymer with a photo-responsive character. Upon light illumination, 3-HF derivatives can be triggered for carbon monoxide (CO) release. The ether linker can also be released emitting formaldehyde (FA). The self-assembly induced micelle can encompass medicine, e.g., doxorubicin (DOX), into it and a controlled release of DOX can be realized upon light illumination. As far as we know, there is no report on the combination donor of CO and DOX and this is the first attempt on the co-release of CO, FA and DOX.

## 1. Introduction

Gas therapy has shown promising applications in disease treatment and is believed to be an alternative treatment modality [1]. The gas molecules are endogenous signaling molecules including nitric oxide (NO), CO, and hydrogen sulfide (H_2_S), which play vital roles in physiological and pathological circumstances. CO is a stable and non-metabolized endogenous signaling molecule. It can promote the mitochondrial respiration inducing more oxygen consumption [2,3] and reactive oxygen species (ROS) production [4,5]. Exogenous CO has also been reported to affect the immune regulation functions by innate immune cells recruitment and myeloid cells differentiation [6]. Moreover, CO can differentially kill cancer cells instead of normal cells presenting little drug resistance [7]. To be noted is that the function of CO is highly concentration dependent, the precise delivery and controlled release are challenging in clinical applications [8]. Therefore, the design of CO controlled release is of important significance.

FA has been commonly applied in cosmetics and food areas and is well known for its antibacterial property [9]. It is also a kind of endogenously reactive carbonyl species and through one-carbon metabolism mechanism, it also enrolls in the synthesis of nucleobases and epigenetic modification [10]. Owing to its strongest activity among all carbonyl species in vivo, aberrant FA concentrations will induce the cross-linking of DNA or proteins, generating genotoxicity or protein malfunction [11,12]. This similar concentration dependent character as endogenous signaling molecule correlates its abnormal concentration with diseases such as cancer, Alzheimer’s disease (AD), diabetes and chronic liver disease [10,13,14,15,16]. The pathological and physiological functions inspire researchers to design innovative FA probes for biological roles and usage investigations. [17,18,19] However, as a potential therapy method, the design of nano-carriers with FA controlled release is still limited [20].

Photo-responsive polymers have received much attention during the past decades. They can be conveniently triggered from outside of the system and controlled from spatial and temporal aspects. Due to the efficient and clean stimulation without any additional chemical reagents, easy modulation by simple changes of wavelength, intensity and illumination time, they have been designed and applied in the biomedical field. [21] To achieve deeper tissue penetration, visible and near-infrared (NIR) light instead of ultraviolet (UV) are adopted to initiate photo-responsive polymers. [22,23] Nano-carriers containing therapeutic agents are designed and show promising applications in disease therapy [24].

The design of controllable release is essential as drugs can be rapidly released from nanoparticles into the target, effectively reducing drug losses. Disulfide bonds reduction in cytoplasm, pH sensitive disassembly in an acidic environment, NIR and ultrasonic irradiation-initiated drugs’ penetration into tissues have been attempted to achieve synergistic effects [25,26,27,28]. Hu’s group has demonstrated the feasibility of a combined release of NO and FA for an antiseptic purpose under light illumination [20]. Simultaneous release of NO and DOX or gentamicin has also been realized by rational molecular design. [29,30] The outcomes indicate an increased antimicrobial property and synergistic effects on antitumor by controlled and simultaneous release of signaling molecules and antibiotics or antitumor agents. However, the combined donor for CO and FA has never been attempted. The co-release of CO and DOX has never been reported either.

**Scheme 1:**
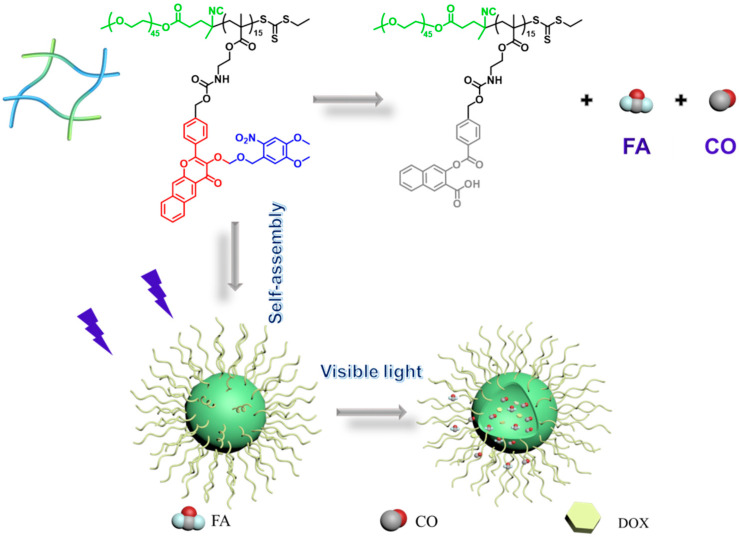
Schematic illustration of the formation of DOX-loaded micelles self-assembled from PEG-b-PFFM diblock copolymers and the photo-triggered co-delivery of CO, FA and DOX.

Therefore, in this work, by rational molecular engineering, a monomer 2-((((4-(3-(((4,5-dimethoxy-2-nitrobenzyl)oxy)methoxy)-4-oxo-4*H*-benzo[*g*]chromen-2-yl)benzyl)oxy)carbonyl)amino)ethyl methacrylate (FFM) containing photo-responsive *o*-nitrobenzyl ether, 3-HF derivatives and ether linker endowing it with photo-responsiveness, CO release and FA release characters was rationally constructed and synthesized. The FFM monomer can be polymerized by a poly (ethylene glycol) (PEG)-based macromolecular initiator similar to the CO-releasing amphiphilic system [31]. Upon assembly, DOX can be loaded into micelles and upon illumination, FA and CO can be released inducing the breakup of micelle structures, and DOX will be simultaneously released (Scheme 1).

## 2. Materials and Methods

### 2.1. Materials

3-hydroxy-2-naphthoic acid, methyl lithium, p-benzaldehyde, acetic acid, acetic anhydride, 6-nitroresveratrol, 6-nitroresveratrol bromine, sulfonyl chloride, doxorubicin hydrochloride (DOX∙HCl) and sodium borohydride (NaBH_4_) were purchased from Sinopharm Chemical Reagent Company (Shanghai, China). Tetramethylazo blue (MTT), ethyl isocyanate methacrylate and deuterated dimethyl sulfoxide (DMSO-d_6_) were purchased from Sigma Aldrich Trading Co., Ltd. (Shanghai, China) and used directly unless otherwise specified. Deuterated chloroform (CDCl_3_) was purchased from Cambridge Isotope Laboratory (Shanghai, China). Organic solvents, including toluene, dichloromethane (DCM), acetonitrile (MeCN), tetrahydrofuran (THF), N, N’-dimethylformylamine (DMF) and dimethyl sulfoxide (DMSO), were purchased from Kelong Chemicals Co., Ltd. (Chengdu, China) and purified in the solvent purification system (Pure Solv^TM^, Milwaukee, WI, USA), and other solvents were used directly. Deionized water was prepared by Milli-Q equipment (Direct-Q3, Merk, Rahway, NJ, USA) and has a specific resistance of 18.2 m Ω· cm^−1^. In total, 10% (*v*/*v*) fetal bovine serum (ExCell Bio, Suzhou, China) and 1% (*v*/*v*) penicillin/Streptomycin Solution purchased from Beyotime biotechnology (Shanghai, China) were added to cell culture medium (DMEM) (Beyotime biotechnology, Shanghai, China). Compound A [31], 1-(chloromethoxymethyl)-4,5-dimethoxy-2-nitrobenzene [32,33] and polyethylene oxide macromolecular chain transfer reagent [34] were synthesized according to the methods reported in the literature.

### 2.2. Instruments and Measurements

Nuclear magnetic resonance (NMR) spectra were measured on a 400 MHz Bruker NMR spectrometer operated in the Fourier transform mode (Bruker, Billerica, MA, USA). High performance liquid chromatography (HPLC) analysis was obtained from Shimadzu HPLC system equipped with an LC-20AP binary pump, an SPD-20A UV-Vis detector, and a Symmetry C18 column. The molecular weight and molecular weight distribution of the polymer were measured by gel permeation chromatography (GPC) (Waters, Waltham, MA, USA) equipped with Waters 1515 solvent delivery pump and Waters 2414 differential refractive index detector (30 °C). The GPC used two linear Styragel columns (HR2 and HR4) in the same series, and the temperature of the column incubator was set to 45 °C. THF of chromatographic grade was used as the mobile phase and the flow rate was set to be 1.0 mL/min. Polystyrene with narrow molecular weight distribution was used as the standard sample for calibration. A dual beam UV–Vis spectrophotometer (Tu-1910, Puxi General Instrument Co., Ltd., Beijing, China) was used to test the UV absorption spectrum. The fluorescence spectrum was tested by fluorescence spectrophotometer (F-4600, Hitachi, Tokyo, Japan). Dynamic laser scattering (DLS) was measured on a laser light scattering instrument (ALV/CGS-3, ALV-Laser Vertriebsgesellschaft m-b.H., Langen, German). Laser (λ_0_ = 632.8 nm) was used as the light source, and scattered light was collected at fixed 90° for 5 min. All data are averaged with three tests. An electrospray ionization mass spectrometry (ESI-MS) (LTQ Orbitrap, Thermo Scientific, Waltham, MA, USA) equipped with an electrospray interface was adopted. The observation and shooting of transmission electron microscope (TEM) were carried out with an electron microscope (JEM-1400 Flash, JEOL Ltd., Tokyo, Japan) at an accelerating voltage of 200 kV. The copper mesh coated by aromatic film and carbon film in turn was used as the carrier, and the dispersion of each assembly was about 20 μL (0.1 g/L) to prepare TEM test samples. The enzyme labeling test of cytotoxicity was carried out on a Thermo Fisher Scientific Instrument (Varioskan Flash, Thermo Fisher Scientific, Waltham, MA, USA).

### 2.3. Synthesis Procedures

#### 2.3.1. Synthesis of Compound B

3-hydroxyflavone derivative A (318 mg, 1 mmol), anhydrous potassium carbonate (150 mg, 1.08 mmol) and 1-(chloromethoxymethyl)-4,5-dimethoxy-2-nitrobenzene (292 mg, 1.12 mmol) were added into a clean round-bottom flask dissolved with 20 mL of DMF. The mixture was stirred at room temperature for 12 h. After the reaction, the filtrate was precipitated in 200 mL of deionized water and filtered. The obtained residue was washed with ethanol to obtain product B (293 mg, yield: 54%). ^1^H NMR (400 MHz, DMSO-d_6_, δ, ppm, Figure S1a): 8.26 (d, J = 8.3 Hz, 1H), 8.10 (d, J = 8.4 Hz, 1H), 7.95 (d, J = 8.2 Hz, 2H), 7.76–7.65 (m, 1H), 7.65–7.55 (m, 2H), 7.37 (d, J = 8.2 Hz, 2H), 5.33 (t, J = 5.6 Hz, 1H), 4.52 (d, J = 5.5 Hz, 2H). ^13^C NMR (101 MHz, CDCl_3_, δ, ppm, Figure S1b): 175.37, 153.82, 151.57, 147.30, 144.59, 138.21, 135.83, 130.50, 130.06, 129.52, 129.06, 128.90, 127.22, 126.89, 126.40, 126.10, 122.96, 114.22, 108.62, 107.53, 95.12, 67.97, 64.77, 56.35. ESI (Figure S1c): *m*/*z* Calc. for C_30_H_25_NO_9_ [M+Na]^+^: 566.1422; found: 566.1427.

#### 2.3.2. Synthesis of FFM Monomer

**Figure 1:**
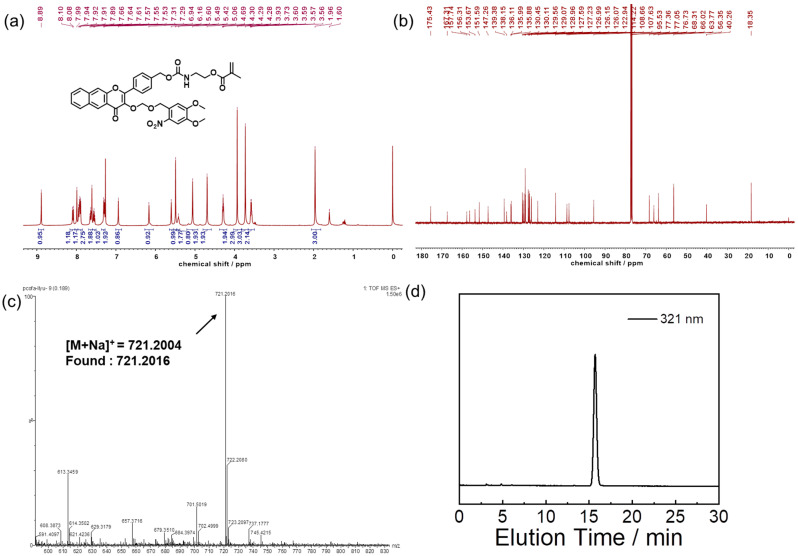
(**a**) ^1^H and (**b**) ^13^C NMR spectra recorded in CDCl_3_ for FFM monomer; (**c**) ESI mass spectrum recorded for FFM monomer; (**d**) HPLC profile recorded at 321 nm for FFM monomer (0.125 mM; CH_3_CN/H_2_O, *v*/*v* = 6/4 as the eluent).

**Figure 2:**
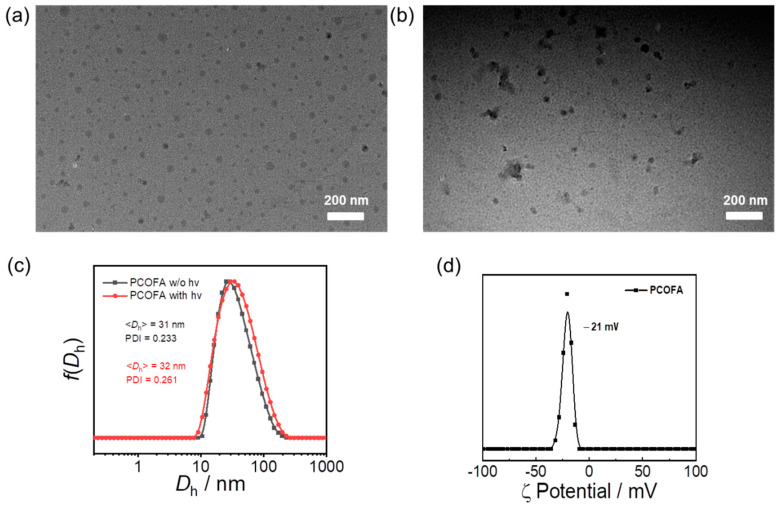
(**a**,**b**) TEM images of aqueous dispersions (0.1 g/L) of PCOFA micelles (**a**) without and (**b**) with irradiation for 2 h; (**c**) intensity-average hydrodynamic distributions of PCOFA micelles without and with light irradiation; (**d**) zeta potential of PCOFA micelles.

Compound B (271 mg, 0.5 mmol) was first mixed with 11 μL dibutyltin dilaurate (DBTL), dissolved in 30 mL of anhydrous toluene and water was removed by azeotropic distillation. A total of 30 mL of dry DMF was added for dissolution and then isocyanoethyl methacrylate (93 mg, 0.6 mmol), also dissolved in 5 mL of dry DMF, was added dropwise into the reaction flask, and stirred overnight at room temperature. The reaction mixture was precipitated into 300 mL deionized water, and the filtered residue was dried in a vacuum drying oven overnight. It was further purified by recrystallization with dichloromethane and n-hexane to obtain FFM monomer (523 mg, yield: 75%). ^1^H NMR (400 MHz, CDCl_3_, δ, ppm, Figure 1a): 8.09 (d, J = 8.3 Hz, 1H), 7.92 (dd, J = 12.4, 8.3 Hz, 4H), 7.69–7.59 (m, 2H), 7.59–7.51 (m, 1H), 7.30 (d, J = 8.1 Hz, 2H), 4.29 (t, J = 5.2 Hz, 2H), 3.58 (q, J = 5.5 Hz, 2H). ^13^C NMR (101 MHz, CDCl_3_, δ, ppm, Figure 1b): 156.31, 153.67, 151.59, 147.26, 138.15, 130.45, 130.11, 129.56, 129.07, 128.96, 127.59, 127.23, 126.99, 126.15, 126.07, 114.22, 108.66, 107.63, 95.53, 68.31, 66.02, 63.77, 56.35, 40.26, 18.35. HPLC analysis (Figure 1c): elution peak at 15.7 min (6:4, *v*/*v*, CH_3_CN/H_2_O; λ = 321 nm). ESI-MS (Figure 2d): *m*/*z* Calc. for C_37_H_34_N_2_O_12_ [M+Na]^+^: 721.2004; found: 721.2016.

#### 2.3.3. Synthesis of PEG45-b-PFFM15 Copolymer (PCOFA)

The diblock copolymer PEG_45_-b-PFFM_15_ was prepared by reversible addition–fragmentation chain-transfer polymerization (RAFT). Briefly, FFM monomer (70 mg, 0.1 mmol), AIBN (0.22 mg, 0.00134 mmol) and macro-graft agent (15 mg, 0.0067 mmol) were dissolved in 140 μL DMSO in the reaction tube equipped with a magnetic stirrer. The reaction tube is degassed through three freeze-thaw cycles, and then sealed under vacuum. After stirring at 70 °C in an oil bath for 10 h, the reaction solution was placed in liquid nitrogen to quench the polymerization reaction. DCM was used for dilution, and the mixture was precipitated in excess ether 3 times. The precipitate was filtered and dried under vacuum overnight to obtain yellow solid PEG_45_-b-PFFM_15_ diblock copolymer (27 mg, yield: 38%). ^1^H NMR (400 MHz, DMSO-d_6_, δ, ppm, Figure S2): 8.70–7.20 (m, 185H), 6.69 (s, 15H), 5.29 (s, 30H), 4.99 (s, 25H), 4.57 (s, 29H), 3.97 (s, 29H), 3.70 (s, 49H), 3.50 (s, 237H). The degree of polymerization (DP) of PCOFA block copolymer was determined to be 15 according to ^1^H NMR analysis (Figure S2). Therefore, the synthesized diblock copolymer is named PEG_45_-b-PFFM_15_. The number average molecular weight (MN) of the diblock copolymer is 5.6 kDa, and the molecular weight distribution (M_w_/M_n_) is determined to be 1.09 (Figure S3).

### 2.4. Self-Assembly of PCOFA Diblock Copolymer

PCOFA diblock copolymer dissolved in THF solution (1 mL, 2 mg/mL) was quickly added to 4 mL deionized water under stirring (1000 rpm/min). The mixture was stirred at room temperature for 30 min. THF was removed by dialysis (MWCO 14 kDa) with deionized water and the aqueous dispersion of the assembly was obtained and stored in the refrigerator.

### 2.5. Characterization of PCOFA Micelles

#### 2.5.1. CMC Test of PCOFA Micelles

Different concentrations (0.2 g/L, 0.1 g/L, 0.05 g/L, 0.025 g/L, 0.0125 g/L, 0.00625 g/L, 0.003125 g/L, 0.0015625 g/L, 0.00078125 g/L and 0.00039062 g/L) of PCOFA loaded with NR and 5 mg/mL of NR in THF solutions were prepared. To each vial 20 μL NR solution was added and dried naturally. The vials were stored in a vacuum drying oven overnight and 1 mL of PCOFA of each concentration was added separately and solubilized for 48 h. Each sample was filtered by 450 nm membrane and fluorescence strength at a wavelength of 636 nm was detected. The CMC value can be obtained from fluorescence intensities changes at 636 nm with PCOFA concentrations (Figure S4).

#### 2.5.2. Optical Properties and Photolysis of PCOFA Micelles 

The degradation process of micelles was monitored by UV–Vis spectroscopy, dynamic light scattering (DLS) and TEM. PCOFA colloidal dispersion (0.1 g/L) was added to a standard quartz cuvette and illuminated with a 410 nm LED lamp (28 mW/cm^2^) for a predetermined time interval to obtain the absorption spectrum of PCOFA colloidal dispersion (Figure S5). The lamp (410 nm, 28 mW/cm^2^) was used throughout the work as light source. The size and distribution of PCOFA colloidal dispersion before and after illumination were measured by DLS, and the morphological changes before and after illumination were observed by TEM (Figure 2).

#### 2.5.3. DOX Loading and Photo-Triggered DOX, FA and CO Release

DOX·HCI (20 mg) and triethylamine (TEA, 6.99 mg) were mixed in 2 mL of DMSO and stirred at room temperature for 4 h for deprotonation and 2 mL of DOX mother liquor with a concentration of 10 mg/mL was obtained for standby. To prepare the DOX-loaded assembly, the initial mass ratio of polymer to DOX is 2:1. A total of 1 mL of THF solution containing 0.2 mg/mL polymer and 0.1 mg/mL DOX was quickly added into 4 mL of water, and stirred at room temperature for half an hour, and then stabilized for half an hour. The dialysis was conducted with deionized water (MWCO 14 kDa) to remove the organic solvent completely. The assembly loaded with DOX was prepared for standby. In total, 1 mL of dialyzed assembly was first freeze-dried and then dissolved in DMSO to test its fluorescence intensity. The content of DOX and the drug loading (DLC) and drug loading rate (DLE) were calculated combined with the standard curve obtained from the fluorescence test of DOX system dissolved in DMSO alone. DLC and DLE are calculated to be 8.2% and 18%, respectively (Figure S9). The release of DOX in response to light is detected by the fluorescence intensity of the dialysates after DOX-loaded micelles with different illumination time at a predetermined time interval were freeze-dried and dialyzed. Specifically, the aqueous dispersion loaded with DOX micelles (800 μL, 0.2 g/L) was divided into three groups and illuminated for 0 min, 10 min and 20 min, respectively. After that, the micelles of different groups were dialyzed with deionized water, and the dialysate was collected according to the predetermined time interval, and new deionized water was added for dialysis. Finally, the collected dialysate was lyophilized and dissolved in DMSO for fluorescence test and analysis. The release rate of DOX was quantitatively obtained by comparing with the fluorescence intensity of the standard curve at 594 nm (565 nm) (Figure 7).

**Figure 3:**
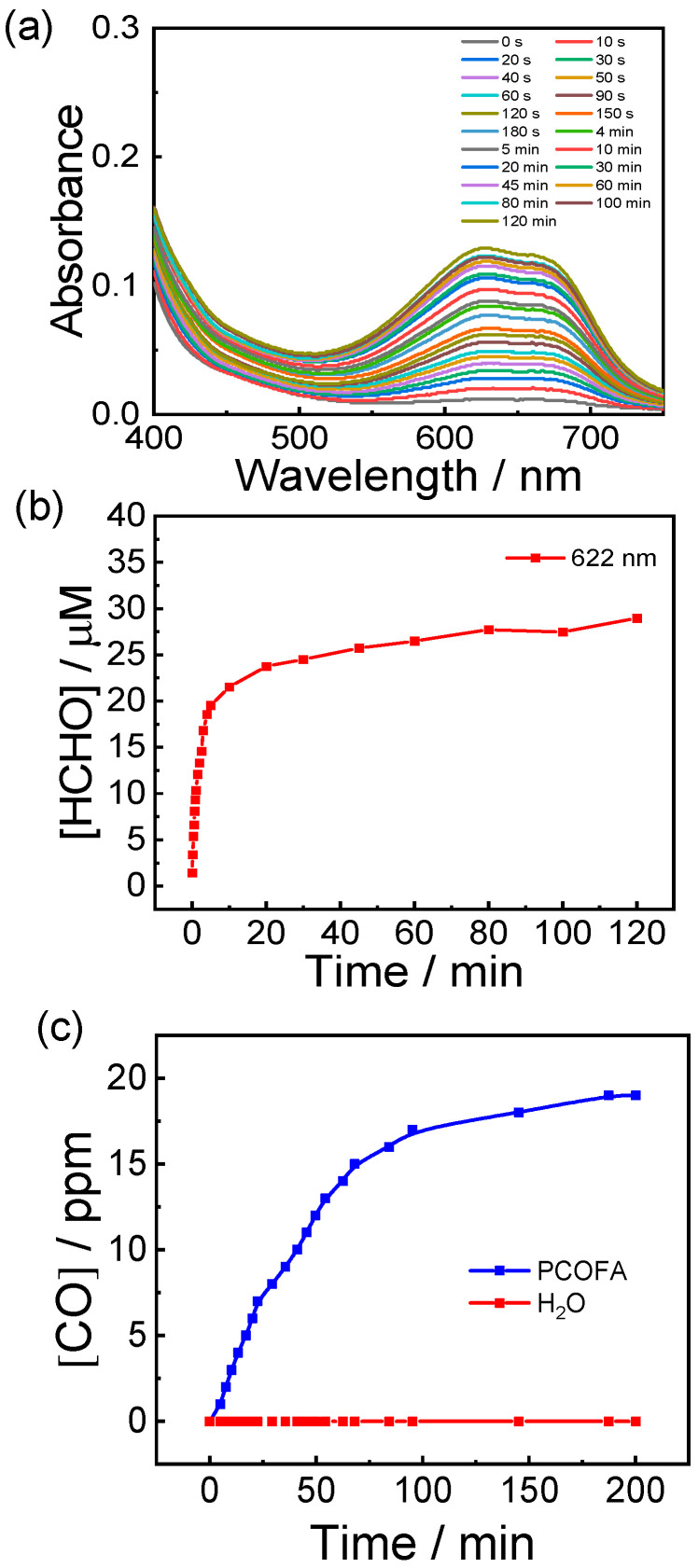
The UV spectra of PCOFA assembly (0.1 g/L diluted to 30.75 μM) with FA test kit (**a**) and characteristic peak intensity at 622 nm change with time (**b**); (**c**) CO release profiles of PCO (0.1 g/L), PCOFA (0.1 g/L) and water under light illumination.

For FA release detection, PCOFA micelles with 30.75 μM and 200 μL were illuminated with at different time intervals and 200 μL of samples from different time intervals were added with an equal volume of 3-methyl-2-benzothiazolinone hydrazone hydrochloride saline compound separately according to the instructions of the formaldehyde detection kit. After that, the mixture was incubated in a water bath at 30 °C in the dark for 20 min. Then, an equal volume of ammonium ferric sulfate solution was added. After incubation in the dark for 10 min, UV absorbance was detected. The formaldehyde emission of PCOFA micelles was quantified by the standard curve established by the absorbance at 622 nm (Figure 3a,b).

Referring to the existing literature, we selected the Anpaer APEG-G gas detector [35] to quantify the CO release after illumination of PCOFA colloidal dispersion. The dispersion of PCOFA assembly (0.1 g/L) was added into the sample bottle with a stirring magnet. The sample bottle was placed together with the CO detector in a closed transparent glass container at a certain distance for illumination. Timing was started when the light source was turned on. Each change of the indicator display was recorded with time until the indicator no longer changed. Assuming that the pressure in the closed container is one atmospheric pressure and the liquid phase and gas phase reach equilibrium, the release of CO can be calculated by the following formula:

Nco = PV_g_/RT + CV_l_ = P(V_g_/RT + V_l_/K)

where N_CO_ is the releasing amount of CO; P is the partial pressure of CO; V_g_ and V_l_ are the volumes of gas phase and liquid phase, 350 mL and 10 mL, respectively; R is the gas constant, 0.0821 L∙atm/(K∙mol); T is the temperature, 298.15 K; C is the concentration of CO in the liquid phase; K is the Henry constant of CO in water. When the temperature is 298.15 K, the Henry constant is 1052.63 L∙atm/mol.

#### 2.5.4. Hemolytic and Cytotoxicity Assessment of DOX-Free and DOX-Loaded Micelles

A total of 2% sheep red blood cells were treated with different concentrations of PCOFA groups (0.0125, 0.025, 0.05, 0.1 mg/mL), Triton X-100 (1%, positive control group) and PBS buffer (negative control group) for the hemolysis test under dark or light conditions. Specifically, sheep red blood cells were centrifuged and washed three times (2000 rpm, 10 min) with PBS buffer (10 mM, pH 7.4), and then mixed with the above groups, respectively. They were incubated together at 37 °C for 1 h, and then centrifuged at 2000 rpm for 10 min. The supernatant after centrifugation was sucked into a 96 well plate, and the hemolysis rate was calculated with the OD value at 567 nm. The hemolysis rate was calculated using the following formula:

Hemolysis% = (A_576, sample_ − A_576, blank_)/(A_576, positive group_ − A_576, blank_) × 100%

A_576, sample_ refers to the absorbance of the supernatant containing micelles at 567 nm; A_576, positive group_ refers to the absorbance of the positive control group at 567 nm; A_576, blank_ refers to the absorbance of the negative control group at 567 nm.

An MTT assay was used to detect the cell survival rate. Human cervical cancer cells (HeLa) and mouse fibroblasts (L929) were selected to test the photo-toxicity under the irradiation and the cytotoxicity of different concentrations of unloaded DOX micelles. Specifically, HeLa and L929 cells were inoculated into 96 well plates with an initial density of 10,000 cells/well, and 100 cells were added 100 μL DMEM medium. After overnight culture, the original medium was replaced with a medium containing PCOFA micelles (0.0125, 0.025, 0.05, 0.1 mg/mL). The control group contained the medium without micelles, while the blank group contained only a medium without cells. After incubation for 4 h, the medium containing micelles was sucked out and PBS buffer was added. One group was illuminated with LED light for 20 min, and the other group was not exposed to light. After illumination, fresh DMEM cell culture medium was replaced, and MTT (10 μL, 5 mg/mL) was added after 24 h of continuous culture solution, then further cultured for 4 h. Then, the suspension was removed and DMSO was added to each well (100 μL/well) and incubated in a constant temperature oscillation chamber at 37 °C for 15 min, and finally the absorbance at 490 nm was recorded with a microplate reader to determine the cell survival rate. The cell photo-toxicity test method is similar to the above. In short, under the irradiation, the groups with different illumination time (0, 10, 20, 30 min) were set to test the survival rate of HeLa cells and L929 cells under this condition. Cell survival was calculated using the following formula:

Cell viability = (A_sample_ − A_blank_)/(A_control_ − A_blank_) × 100%

A_sample_ refers to the absorbance measured by the hole in which micelles are co-incubated with cells; A_blank_ refers to the absorbance measured only in the pores of cells without micelles; A_control_ refers to the absorbance measured after adding MTT to the culture medium without cells. Each experiment was conducted in six groups in parallel, and the data were displayed as mean plus standard deviation (SD).

### 2.6. Statistical Analysis

Mean ± standard deviations were adopted for data presentation and Prism 8.0 software (GraphPad, San Diego, CA, USA) and student’s t-test were adopted for analysis. 

## 3. Results

### 3.1. Design and Characterization of CO, FA, and COX Co-Release Diblock Copolymer

**Scheme 2:**
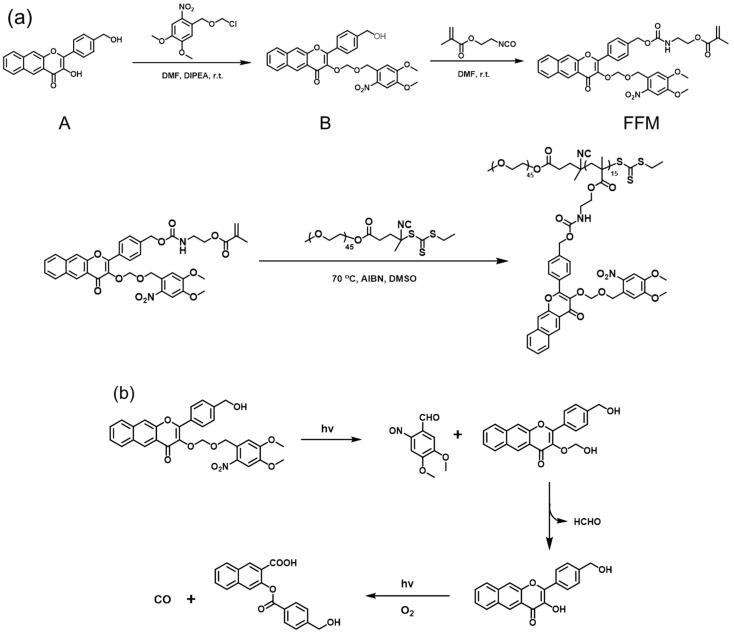
(**a**) Synthetic routes of CO, FA and DOX co-releasing FFM monomer and a PCOFA amphiphilic diblock copolymer; (**b**) proposed photolysis mechanism of compound B under visible light irradiation.

It was reported that 3-HF- derivative can be utilized as a potential CO donor [36,37,38,39,40]. Hu’s group [31] has advocated and applied 3-HF into the construction of a non-metal CO-releasing micelle via RAFT. In another work, 5-hydroxy-2-nitrobenzaldehyde was reacted with 1-((chloromethoxy)methyl)-2-nitrobenzene to generate an FA donor [21]. Inspired by these designs, a 3-HF derivative (A) was first reacted with 1-((chloromethoxy)methyl)-4,5-dimethoxy-2-nitrobenzene to form 3-(((4,5-dimethoxy-2-nitrobenzyl)oxy)methoxy)-2-(4-(hydroxymethyl)phenyl)-4H-benzo[g]chromen-4-one (B) (Scheme 2a) containing three photo-responsive moieties (i.e., 2-nitobenzyl ether, 3-HF derivative and FA released ether bond) within the same molecule. Then, the hydroxyl group on a benzene ring was reacted with the isocyanate group to generate methacrylated B named as an FFM monomer. The obtained FFM monomer can be polymerized by RAFT to form an amphiphilic diblock copolymer of PEO-b-PFFM (PCOFA) by a PEO-based macroinitiator.

The structure of the FFM monomer was confirmed by NMR, HPLC and high-resolution mass spectroscopy (HRMS) (Figure 1). The degree of polymerization of PFFM in PCOFA and PFM in PCO were calculated to be 15 and 13 by NMR (Figure S2). The molecular weights of PCOFA were calculated to be 5.6 kDa (M_w_/M_n_ = 1.07) by GPC (Figure S3). The critical micellization concentration (CMC) of the PCOFA diblock copolymer was determined to be 21 mg/L (Figure S4). TEM was used to test the morphology of PCOFA assembly and the pictures present spherical micellar nanoparticles with diameters of 23 nm (Figure 2a) akin to the observation in DLS (Figure 2c and Figure S10). The prepared PCOFA micelles were negatively charged in an aqueous solution with a zeta potential of −21 mV (Figure 2d).

### 3.2. Visible Light-Triggered CO, FA and Mechanism

PCOFA micelles, after illumination with a 410 nm LED lamp (28 mW/cm^2^) for 2 h, show no obvious change in the morphology of micellar nanoparticles (Figure 2a,b). DLS was also used to monitor the diameter changes of micellar nanoparticles before and after illumination. The average hydration diameters before and after illumination were ~31 nm, which were consistent with TEM. The diameter of micellar nanoparticles did not change significantly with the extension of illumination time (Figure 2c). This may be due to the insoluble character of degraded products and the hydrophilic end of PEG, which causes the micelles to still maintain their original shape after illumination.

The 3-HF derivative was designed as the basic structure and the side chain bearing light sensitive *o*-nitrobenzyl group was reacted to form ether so as to protect the phenolic hydroxyl groups and endow the possibility of macromolecules formation by free radical polymerization. The 3-HF derivative itself can release CO under light triggering, and the semi-acetal structure is designed into the *o*-nitrobenzyl group. After ether formation, the unstable semi-acetal structure is skillfully exposed under visible light to release FA, and then the unprotected 3-HF derivative is continuously illuminated to release CO. This means that the functional units of light triggered co-release of CO and FA can be polymerized and introduced into the polymer, so that the obtained PCOFA diblock polymer can simultaneously release CO and FA under light in a certain wavelength range. Based on this design scheme, the light response behaviors of small donor molecules (compound B, Scheme 2a) and PCOFA diblock polymers under light were studied. It is worth noting that the water solubility of compound B is lower compared with unmodified 3-HF derivative precursors, which may be due to the hydrophobicity of *o*-nitrobenzyl groups. Because of the poor solubility of compound B in water, we chose the mixed solvent of DMSO and water (*v*/*v*, 8/2) for spectral study. As shown in Figure S5a, compound B is responsive to visible light, which is confirmed by the change of UV absorption spectrum with the extension of illumination time. The main absorption wavelengths of compound B are concentrated between 260 nm and 500 nm, among which 270 nm, 335 nm and 385 nm are the main absorption characteristic peaks (Figure S5b). Within 10 min of irradiation, the UV absorption value changes rapidly, and the absorption intensity of the three main absorption characteristic peaks decreases with the increase of illumination time. After 10 min of irradiation, the absorption spectrum of compound B gradually reaches equilibrium. In addition, we also tested the fluorescence emission spectrum of compound B, as shown in Figure S5c,d, and weak fluorescence at 493 nm before illumination was observed. In the first 50 s of illumination, the fluorescence intensity of compound B at 607 nm increased significantly (Figure S5c), which corresponds to the breaking of the ether bond between *o*-nitrobenzyl group and 3-HF at the beginning of illumination, resulting in the hydroxyl exposure of 3-HF. After that, the illumination time continued to be extended, and the fluorescence intensity at 607 nm gradually decreased until it was too weak to be detected. In this process, the exposed 3-HF continued to undergo light reaction to generate the by-product 3-((4-(hydroxymethyl)benzoyl)oxy)-2-naphthoic acid (Figure S5d) and release CO. From the optical spectra measured above, it can be concluded that the designed donor molecular compound B combines two light response parts into one molecule, and the change of fluorescence can be used to track the release of FA and CO through the unique excited state intramolecular proton transfer (ESIPT) properties of 3-HF derivatives.

In order to verify whether the prepared PCOFA diblock copolymer has the same light response behavior as small molecules, the changes of UV and fluorescence spectra of PCOFA aqueous solution under the same light intensity were also tested. Considering that the light sensitive units that can release CO and FA are wrapped in the micelle core, a longer irradiation time for spectral recording was used. As shown in Figure S6a, the profile of the main characteristic UV absorption peaks at 270 nm and 335 nm of the aqueous solution of PCOFA assembly is similar to that of compound B with the extension of illumination time with the absorption intensity decreasing gradually. However, compared with the variation range of compound B, the decrease of UV absorption intensity of PCOFA micellar nanoparticles changed more slowly with the illumination time, and gradually tended to balance after about 20 min of irradiation (Figure S6b). The UV spectrum of PCOFA micelle particles proves that the aqueous solution of PCOFA micelles does break the chain and has light response behavior under the irradiation of light. On the one hand, the slow changing rate may be due to the hydrophobicity of the micelle core, which makes it difficult for water molecules to enter the micelle, resulting in the poor dissolution of nano-micelles. It is speculated that the PCOFA micellar nanoparticles exist in the form of micellar clusters in an aqueous environment, resulting in the limited movement of macromolecular chains. Therefore, the photo-degradation rates of small molecules and macromolecular nano-micelles will be different under the same light conditions. From the UV spectra of compound B and PCOFA micelles above, PCOFA micelles have the same response behavior to light as small molecules, but their polymer chains are not completely dissociated into single chains during photolysis, which can also be proved by the results of DLS (Figure 2b) and TEM (Figure 2c). The fluorescence emission spectrum of PCOFA assembly in aqueous solution was monitored. Due to the unique ESIPT properties of flavonoid derivatives, the fluorescence emission spectrum of PCOFA aqueous solution is similar to that of compound B, with double fluorescence emission wavelengths. The change is mainly in two stages (Figure S6c). The fluorescence emission intensity at 605 nm first increases and then decreases, and the fluorescence intensity at 475 nm continues to decrease. That is, the transition from blue light to red light in the first 12 s and the change from red light to colorless under continuous light for the next 60 min (Figure S6d). The photo-triggered fluorescence transition is also consistent with the change of compound B precursor, indicating that the formation of micellar nanoparticles in an aqueous solution will not change the light reaction pathway. However, compared with the precursor B in the mixed solvent, the fluorescence decay rate of micellar nanoparticles in a pure aqueous solution seems to be slower, which is quite different from the fluorescence emission spectrum of compound B. The fluorescence intensity of PCOFA micelles at 475 nm is stronger than that at 607 nm at the beginning, and compared with the emission intensity of compound B, the intensity change at 607 nm is very small and not obvious with the extension of illumination time. This result not only reveals that the photolysis process is highly correlated with the aggregation state of 3-HF in donor molecules, but also provides a feasible strategy for regulating the release behavior of CO.

Under 410 nm visible light irradiation (28 mW/cm^2^), the ether bond of *o*-nitrobenzyl group breaks, exposing the semi-acetal structure. The unstable structure will spontaneously release FA, and the unprotected 3-HF derivative will undergo photo-oxidation under the continuous irradiation of visible light, and then release CO. In order to further verify that the prepared PCOFA diblock copolymer can be used as the donor polymer of FA and CO, the release of FA in the aqueous solution of PCOFA assembly was quantified by FA kit. The release of CO was quantified by portable CO detector. As shown in Figure 3a,b, the UV absorption intensity of 30 μM PCOFA assembly increased rapidly within 20 min of illumination and gradually reached equilibrium at about 120 min, which indicates the release equilibrium of FA at 2 h. The change of UV absorption intensity at 622 nm was measured by reacting FA aqueous solution of different concentrations with FA kit, and the standard curve was obtained by linear fitting (Figure S7a,b). It is inferred that 30 μM PCOFA micelles can release 30 μM CO from the standard curve corresponding to 1 equivalent polymer to CO. A portable CO detector was used to monitor the CO release in the aqueous solution of PCOFA assembly. As shown in Figure 3c, the release of CO in the aqueous solution of PCOFA assembly gradually increases with the extension of illumination time and is stable after 200 min of irradiation. When the aqueous solution of PCOFA assembly tends to reach equilibrium, it can release ~19 ppm CO. The monitoring results combined with the existing literature reports prove that the controlled release of CO by adjusting the light source intensity and illumination time can be realized in this system. Overall, the release monitoring experiments of FA and CO confirmed that PCOFA micelles can be used as polymer donors for light-triggered co-delivery of CO and FA.

**Figure 4:**
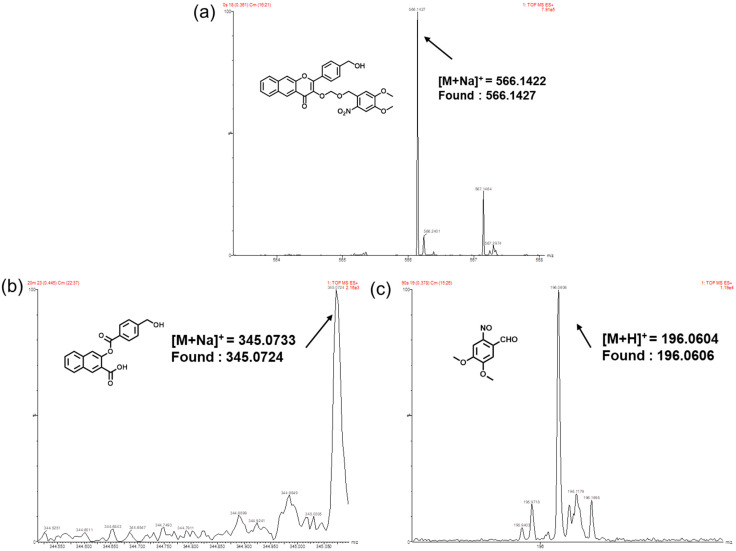
ESI mass spectra recorded for compound B without (**a**) or with (**b**,**c**) irradiation for 20 min.

To unravel the mechanism of photo-triggered CO and FA release, HRMS was used to identify fragments with or without light illumination. As shown in Scheme 2b, the main ionic peaks of compound B, 4,5-dimethoxy-2-nitrobenzaldehyde and 3-(benzoyloxy)-2-naphthoic acid derivatives were successfully detected. It corresponds to the product of FA released by ether bond breaking of compound B after illumination in Scheme 2b and the main by-product of CO released by photo-oxidation after continuous illumination (Figure 4b,c). The above mass spectrometry results are consistent with the reported photo-degradation process of 3-HF derivatives as CO donor molecules. [32] Figure 4a shows that only the ionic peak of compound B is found by mass spectrometry analysis without light. These outcomes not only prove the good stability of the donor molecules under dark conditions, but also can photo-responsively co-deliver CO and FA. Because compound B is the structure unit of PCOFA, it can therefore be concluded that the diblock polymer can be degraded and release FA and CO under light illumination.

### 3.3. Co-Release of DOX, CO and FA for Antitumor Attempt

**Figure 5:**
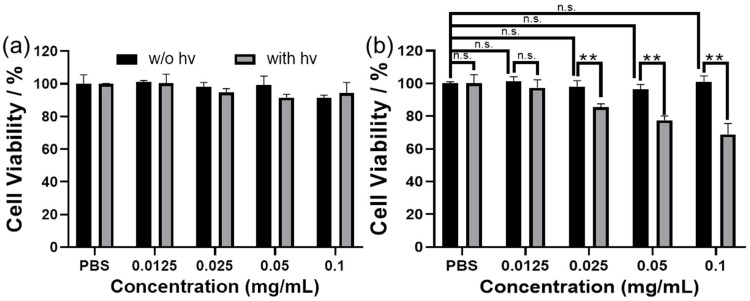
Cell viability of (**a**) L929 and (**b**) HeLa at different PCOFA micelle concentrations (0.0125 mg/mL, 0.025 mg/mL, 0.05 mg/mL, 0.1 mg/mL) without DOX with or without irradiation for 20 min after 24 h incubation. Data are presented as mean ± s.d. (*n* = 3); n.s., not significant; *p* values were calculated in comparison with the non-irradiated groups. ** *p* < 0.01.

**Figure 6:**
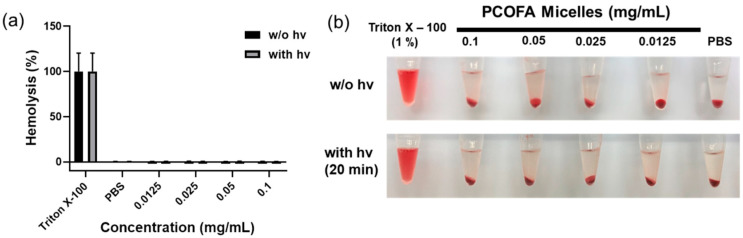
(**a**) Quantification and (**b**) macroscopic images of hemolysis of sheep erythrocytes (2%) treated with Triton X-100 (1%, positive control), PBS (negative control) and PCOFA micelles at different concentrations without or with light irradiation for 20 min.

Before evaluating the effect of PCOFA micelles on tumor cells, cytotoxicity and cell hemolysis experiments were conducted. The determination of illumination time is based on a cell photo-toxicity experiment to ensure that the survival rate of cells is not affected by the toxicity of illumination itself. As shown in Figure S8, in the absence of PCOFA micellar nanoparticles, the cell viability did not decrease significantly under light irradiation for 20 min, but drop significantly when subjected to a longer irradiation time. Therefore, the illumination time was determined to be 20 min. A cell cytotoxicity experiment reveals that PCOFA micelles without light illumination show no killing effects on cells at tested concentrations. However, after incubation with HeLa cells for 24 h under illumination for 20 min, PCOFA micelles showed an enhanced inhibitory effect on HeLa cells with the increase of concentration. When the concentration of PCOFA micelles was 0.1 g/L, the activity of HeLa cells decreased to ~60% after illumination (Figure 5). In addition, ideal anticancer drugs can selectively kill cancer cells without damaging normal mammalian cells. Therefore, the effect of micellar nanoparticles on L929 cells was tested. As shown in Figure 5a, PCOFA micelles have no obvious toxicity on normal mammalian cells. The maximum concentration of PCOFA micelles is determined by a cell hemolysis experiment. When the concentration of PCOFA micelles is 0.1 g/L, it does not show obvious hemolysis in Figure 6. It is, therefore, speculated that the increase of cytotoxicity may come from the synergistic anticancer effect of CO and FA released by light after PCOFA micelles are ingested by cells. Photo-responsive PCOFA nano-micelles are promising for practical anticancer therapy. At the same time, they can be considered as nano-carriers to deliver other therapeutic reagents.

**Figure 7:**
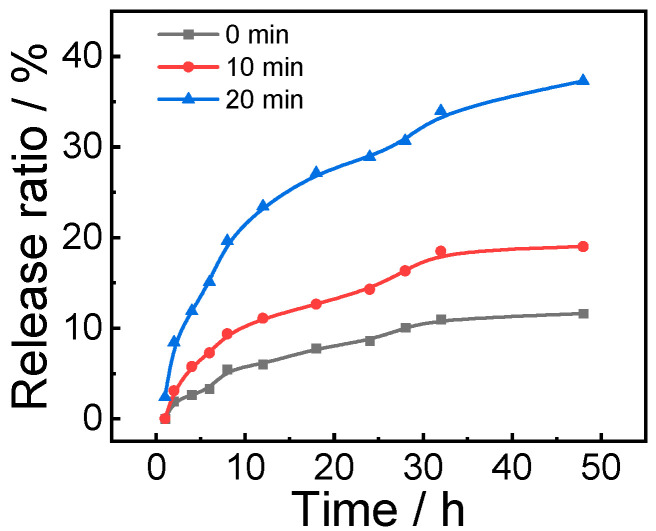
DOX releasing profile of PCOFA micelles under light illumination at different time intervals (0, 10 and 20 min).

Considering that PCOFA can self-assemble to form nano-micelles and has certain inhibitory effects on tumor cells, the nano-micelles can be used to load other anticancer drugs for synergistic treatment to overcome the limitations of single treatment. The anticancer drug DOX was loaded into PCOFA micelles to further evaluate the synergistic anticancer effect. The behavior of DOX release from PCOFA nano-micelles loaded with DOX was first tested. The experimental results show that the release rate of DOX can be adjusted by the time of light irradiation. The drug-loaded micelles had ~11.6% DOX release within 48 h without light, and the drug-loaded micelles exposed to light for 10 min and 20 min had 18.9% and 37.3% DOX release, respectively (Figure 7). Drugs within micelles can be released with time. Moreover, the hydrophobic interactions among drugs loaded in micelles will further hamper the release rate with time presenting a controlled release manner.

**Figure 8:**
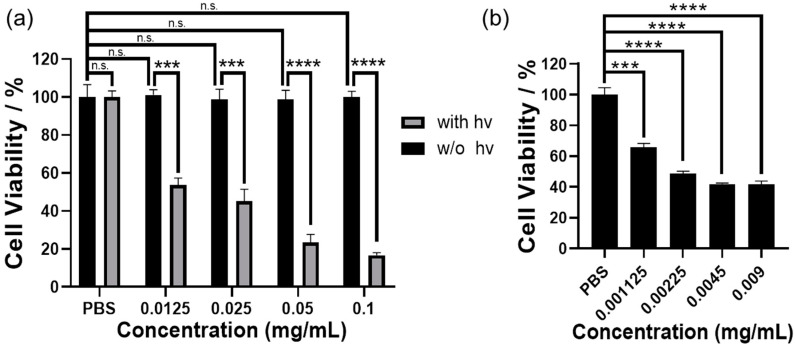
(**a**) Cell viability of DOX-loaded PCOFA micelles with HeLa cells for 24 h with or without light irradiation for 20 min at varying concentrations (0.0125 mg/mL, 0.025 mg/mL, 0.05 mg/mL, 0.1 mg/mL); (**b**) cell viability of HeLa cells with DOX at different concentrations (1.125, 2.25, 4.5, 9 μg/mL) for 24 h. *** *p* < 0.001; **** *p* < 0.0001.

Then, the antitumor effect of DOX-loaded PCOFA micelle was tested by co-incubation with HeLa cells. Specifically, HeLa cells incubated with DOX-loaded PCOFA micelles for 24 h were irradiated with or without light for 20 min, and the cell survival rate was tested by MTT method. As shown in Figure 8, DOX-loaded PCOFA micelles show negligible cytotoxicity in the absence of light, while the mortality of HeLa cells increased with the increase of the concentration of DOX-loaded PCOFA nano-micelles under light illumination. When the concentration of DOX-loaded PCOFA micelles was 0.1 g/L, the cell mortality under light illumination was as high as ~83.5%, showing an increase of ~52% compared with simple DOX-loaded PCOFA nano-micelles. The DOX alone, however, can only induce ~42% mortality (Figure 8b). The concentration of DOX in DOX-loaded micelles was determined to be 0.018 mg/mL, and the drug-loading capacity (DLC) and drug-loading efficiency (DLE) were calculated to be 8.2% and 18%, respectively (Figure S9). In conclusion, the experimental results show that the nano-micelles formed by polymer self-assembly that can co-release two gas molecules (CO and FA) have a better inhibitory effect on tumor cells than PCOFA micelles without DOX and DOX alone. This strategy achieves the purpose of the combination of gas therapy and drug delivery, overcoming the limitations of single treatment. This may provide a new idea of co-delivery of two gas molecules and drug molecules in anticancer therapy.

## 4. Conclusions

In summary, polymer micelles with drug loading and releasing and co-delivery of CO and FA gases under light irradiation have been rationally designed and synthesized. Through molecular design, the synthesized molecule can bear CO and FA donors within the molecular structure. Utilizing a macromolecular initiator, a PCOFA polymer strand can be obtained and further self-assemble into micelles. Pharmaceutical molecules can be loaded into micelles through the assembly process. Upon light illumination, the co-release of CO and FA will change the structure of micelles, leading to medicine release and eventually realizing the co-release of both gases and medicine molecules. The nano-carriers present good compatibility with negligible effect on normal cells and show higher anti-cancer efficiencies than pure DOX or DOX-loaded PCOFA without a light irradiation system. This design may provide a way for future utilization of gaseous molecule-based therapy and a combination of two or more ways to overcome the limitations of single treatment.

## Data Availability

Data are available in a publicly accessible repository.

## Supplementary Materials

The following supporting information can be downloaded at: https://www.mdpi.com/article/10.3390/polym14122416/s1, Figure S1 (a) ^1^H NMR spectra recorded in DMSO-d_6_ for compound A; (b) ^13^C NMR spectra rec-orded in DMSO-d_6_ for compound A; (c) ESI mass spectrum recorded for compound A in acetonitrile. Figure S2 ^1^H NMR spectra recorded in DMSO-d_6_ for PEO_45_-b-PFFM_15_ (PCOFA). Figure S3 GPC Elution profiles of PEG_45_-based macroinitiator and PCOFA. Figure S4 Concentration-dependent changes of surface tension of aqueous dispersions of PCOFA diblock copolymer. Figure S5 UV absorption spectra (a) and trend diagram of main characteristic absorption peaks with illumination time (b) of Compound B (80 μM, DMSO/H_2_O, *v*/*v* = 8/2) irradiated by 410 nm LED lamp (28 mW/cm^2^). The fluorescence emission spectrum of B from 0–50 s (c) and 60s-90s (d) (λex = 405 nm; slit widths: E_x._ = 5.0 nm, E_m._ = 5.0 nm). Figure S6 UV absorption spectra (a) and trend profile of main characteristic absorption peaks with illumination time (b) of PCOFA micelle solution (0.1 g/L) irradiated by 410 nm LED lamp (28 mW/cm^2^). The fluorescence emission spectrum (c) and trend profile of main characteristic absorption peaks with illumination time (d) of PCOFA micelle solution (0.1 g/L) (λ_ex_ = 405 nm; slit widths: E_x._ = 5.0 nm, E_m._ = 5.0 nm). Figure S7 UV absorption spectra of (a) different concentrations of FA in water and (b) linear fitting standard curve at 622 nm. Figure S8 Cell viability of (a) HeLa and (b) L929 under 410 nm irradiation (28 mW/cm^2^) for varying duration after 24 h incubation. Data are presented as means s.d. (n = 3); n.s., not significant; *p* values were calculated in comparison with the non-irradiated groups. * *p* < 0.05; ** *p* < 0.01. Figure S9 (a) Fluorescent spectra of DOX at different concentrations in DOMSO (λ_ex_= 480 nm; slit widths: E_x._ = 10 nm, E_m._ = 10 nm) (b) Fitting curve of DOX concentration and fluorescence intensity at 565 nm. Figure S10 Number-average hydrodynamic distributions of PCOFA micelles without and with light irradiation. In all cases, the irradiation wavelength was 410 nm and the intensity was 28 mW/cm^2^.

## References

[1] Yu L.D., Hu P., Chen Y. (2018). Gas-Generating Nanoplatforms: Material chemistry, multifunctionality, and gas therapy. Adv. Mater..

[2] Morse D., Pischke S.E., Zhou Z.H., Davis R.J., Flavell R.A., Loop T., Otterbein S.L., Otterbein L.E., Choi A.M.K. (2003). Suppression of inflammatory cytokine production by carbon monoxide involves the JNK pathway and AP-1. J. Biol. Chem..

[3] Zuckerbraun B.S., Chin B.Y., Bilban M., d’Avila J.C., Rao J., Billiar T.R., Otterbein L.E. (2007). Carbon monoxide signals via inhibition of cytochrome c oxidase and generation of mitochondrial reactive oxygen species. FASEB J..

[4] Wegiel B., Gallo D., Csizmadia E., Harris C., Belcher J., Vercellotti G.M., Penacho N., Seth P., Sukhatme V., Ahmed A. (2013). Carbon monoxide expedites metabolic exhaustion to inhibit tumor growth. Cancer Res..

[5] Manoharan D., Li W.P., Yeh C.S. (2019). Advances in controlled gas-releasing nanomaterials for therapeutic applications. Nanoscale Horiz..

[6] Motterlini R., Otterbein L.E. (2010). The therapeutic potential of carbon monoxide. Nat. Rev. Drug Discov..

[7] Szabo C. (2016). Gasotransmitters in cancer: From pathophysiology to experimental therapy. Nat. Rev. Drug Discov..

[8] Garcia-Gallego S., Bernardes G.J.L. (2014). Carbon-Monoxide-Releasing Molecules for the delivery of therapeutic CO in vivo. Angew. Chem. Int. Ed..

[9] McDonnell G., Russell A.D. (1999). Antiseptics and disinfectants: Activity, action, and resistance. Clin. Microbiol. Rev..

[10] Burgos-Barragan G., Wit N., Meiser J., Dingler F.A., Pietzke M., Mulderrig L., Pontel L.B., Rosado I.V., Brewer T.F., Cordell R.L. (2017). Mammals divert endogenous genotoxic formaldehyde into one-carbon metabolism. Nature.

[11] Lai Y.Q., Yu R., Hartwell H.J., Moeller B.C., Bodnar W.M., Swenberg J.A. (2016). Measurement of endogenous versus exogenous formaldehyde-induced DNA-protein crosslinks in animal tissues by stable isotope labeling and ultrasensitive mass spectrometry. Cancer Res..

[12] Pontel L.B., Rosado I.V., Burgos-Barragan G., Garaycoechea J.I., Yu R., Arends M.J., Chandrasekaran G., Broecker V., Wei W., Liu L.M. (2015). Endogenous formaldehyde is a hematopoietic stem cell genotoxin and metabolic carcinogen. Mol. Cell..

[13] Smaga L.P., Pino N.W., Ibarra G.E., Krishnamurthy V., Chan J. (2020). A photoactivatable formaldehyde donor with fluorescence monitoring reveals threshold to arrest cell migration. J. Am. Chem. Soc..

[14] Tong Z.Q., Zhang J.L., Luo W.H., Wang W.S., Li F.X., Li H., Luo H.J., Lu J., Zhou J.N., Wan Y. (2011). Urine formaldehyde level is inversely correlated to mini mental state examination scores in senile dementia. Neurobiol. Aging.

[15] Yu P.H., Zuo D.M. (1993). Oxidative deamination of methylamine by semicarbazide-sensitive amine oxidase leads to cytotoxic damage in endothelial-cells-possible consequences for diabetes. Diabetes.

[16] Baan R., Grosse Y., Straif K., Secretan B., Ghissassi F.E., Bouvard V., Benbrahim-Tallaa L., Guha N., Freeman C., Galichet L. (2009). A review of human carcinogens--Part F: Chemical agents and related occupations. Lancet Oncol..

[17] Tao R.R., Liao M.H., Wang Y.X., Wang H., Tan Y.H., Qin S.Y., Wei W.J., Tang C.Z., Liang X.G., Han Y.F. (2022). In situ imaging of formaldehyde in live mice with high spatiotemporal resolution reveals aldehyde dehydrogenase-2 as a potential target for Alzheimer’s disease treatment. Anal. Chem..

[18] Huang S.M., Li Z.J., Liu M.H., Zhou M.J., Weng J.T., He Y., Jiang Y., Zhang H.T., Sun H.Y. (2022). Reaction-based fluorescent and chemiluminescent probes for formaldehyde detection and imaging. Chem. Commun..

[19] Du Y.M., Zhang Y.Q., Huang M.R., Wang S.S., Wang J.Z., Liao K.K., Wu X.J., Zhou Q., Zhang X.H., Wu Y.D. (2021). Systematic investigation of the aza-Cope reaction for fluorescence imaging of formaldehyde in vitro and in vivo. Chem. Sci..

[20] Duan Y.T., He K.W., Zhang G.Y., Hu J.M. (2021). Photoresponsive micelles enabling codelivery of nitric oxide and formaldehyde for combinatorial antibacterial applications. Biomacromolecules.

[21] Huang Y., Dong R.J., Zhu X.Y., Yan D.Y. (2014). Photo-responsive polymeric micelles. Soft Matter..

[22] Viger M.L., Grossman M., Fomina N., Almutairi A. (2013). Low power upconverted near-IR light for efficient polymeric nanoparticle degradation and cargo release. Adv. Mater..

[23] Palao E., Slanina T., Muchova L., Solomek T., Vitek L., Klan P. (2016). Transition-metal-free CO-releasing BODIPY derivatives activatable by visible to NIR light as promising bioactive molecules. J. Am. Chem. Soc..

[24] Guo R.R., Tian Y., Wang Y.J., Yang W.L. (2017). Near-infrared laser-triggered nitric oxide nanogenerators for the reversal of multidrug resistance in cancer. Adv. Funct. Mater..

[25] Wang W.W., Cheng D., Gong F.M., Miao X.M., Shuai X.T. (2012). Design of Multifunctional micelle for tumor-targeted intracellular drug release and fluorescent imaging. Adv. Mater..

[26] Dai J., Lin S.D., Cheng D., Zou S.Y., Shuai X.T. (2011). Interlayer-crosslinked micelle with partially hydrated core showing reduction and pH dual sensitivity for pinpointed intracellular drug release. Angew. Chem. Int. Ed..

[27] Xiao H., Li X.X., Zheng C.J., Liu Q.M., Sun C.Y., Huang J.S., Wang Y., Yuan Y.Y. (2020). Intracellular pH-responsive polymeric micelle for simultaneous chemotherapy and MR imaging of hepatocellular carcinoma. J. Nanopart. Res..

[28] Yin T.H., Wang P., Li J.G., Zheng R.Q., Zheng B.W., Cheng D., Li R.T., Lai J.Y., Shuai X.T. (2013). Ultrasound-sensitive siRNA-loaded nanobubbles formed by hetero-assembly of polymeric micelles and liposomes and their therapeutic effect in gliomas. Biomaterials.

[29] Ding Z.L., He K.W., Duan Y.T., Shen Z.Q., Cheng J., Zhang G.Y., Hu J.M. (2020). Photo-degradable micelles for co-delivery of nitric oxide and doxorubicin. J. Mater. Chem. B.

[30] Duan Y.T., Zhang M.Y., Shen Z.Q., Zhang M.D., Zheng B., Cheng S., Hu J.M. (2021). Photoresponsive Vesicles Enabling Sequential Release of Nitric Oxide (NO) and Gentamicin for Efficient Biofilm Eradication. Macromol. Rapid Commun..

[31] Cheng J., Zheng B., Cheng S., Zhang G.Y., Hu J.M. (2020). Metal-free carbon monoxide-releasing micelles undergo tandem photochemical reactions for cutaneous wound healing. Chem. Sci..

[32] Yong P.K., Banerjee A. (2005). Photochemistry of 2-Nitrobenzyl Enol Ethers: Oxidative CC Bond Scission. Org. Lett..

[33] Hansen D.A., Koch A.A., Sherman D.H. (2015). Substrate Controlled Divergence in Polyketide Synthase Catalysis. J. Am. Chem. Soc..

[34] Che H.L., Cao S.P., Van Hest J.C.M. (2018). Feedback-induced temporal control of “breathing” polymersomes to create self-adaptive nanoreactors. J. Am. Chem. Soc..

[35] Hasegawa U., Van Der Vlies A.J., Simeoni E., Wandrey C., Hubbell J.A. (2010). Carbon monoxide-releasing micelles for immuno-therapy. J. Am. Chem. Soc..

[36] Anderson S.N., Richards J.M., Esquer H.J., Benninghoff A.D., Arif A.M., Berreau L.M. (2015). A structurally-tunable 3-Hydroxyflavone motif for visible light-induced carbon monoxide-releasing molecules (CORMs). ChemistryOpen.

[37] Soboleva T., Berreau L.M. (2019). Tracking CO Release in Cells via the Luminescence of Donor Molecules and/or their By-Products. Isr. J. Chem..

[38] Popova M., Soboleva T., Ayad S., Benninghoff A.D., Berreau L.M. (2018). Visible-light-activated quinolone carbon-monoxide-releasing molecule: Prodrug and albumin-assisted delivery enables anticancer and potent anti-inflammatory effects. J. Am. Chem. Soc..

[39] Soboleva T., Esquer H.J., Benninghoff A.D., Berreau L.M. (2017). Sense and release: A thiol-responsive flavonol-based photonically driven carbon monoxide-releasing molecule that operates via a multiple-input AND logic gate. J. Am. Chem. Soc..

[40] Soboleva T., Benninghoff A.D., Berreau L.M. (2017). An H_2_S-sensing/CO-releasing flavonol that operates via logic gates. Chempluschem.

