# Supplementary material for: Photo-Responsive Micelles with Controllable and Co-Release of Carbon Monoxide, Formaldehyde and Doxorubicin
Source: Polymers (Basel). 2022 Jun 14;14(12):2416. doi: 10.3390/polym14122416 (PMC9230906; doi:10.3390/polym14122416)
Supplement: Supplementary file 1 [file polymers-14-02416-s001.zip › polymers-1742848-supplementary.pdf]

Photo-responsive micelles with controllable and co-release of carbon monoxide, formaldehyde and doxorubicin

Bin Zheng, [*a§] **Lulu Yu,**[b§] **Huaze Dong** [a], **Jinmiao Zhu** [a], **Liang Yang** [a] **and Xinsong Yuan** [a],

a. School of Chemistry and Chemical Engineering, Hefei Normal University, Hefei, Anhui 230061, P.R. China. E-mail: zhengbin@hfnu.edu.cn; Tel:+86-551-63758370

b. Department of Polymer Science and Engineering, University of Science and Technology of China, Hefei, Anhui 230026, P. R. China.

§ The two contribute equally to the work.

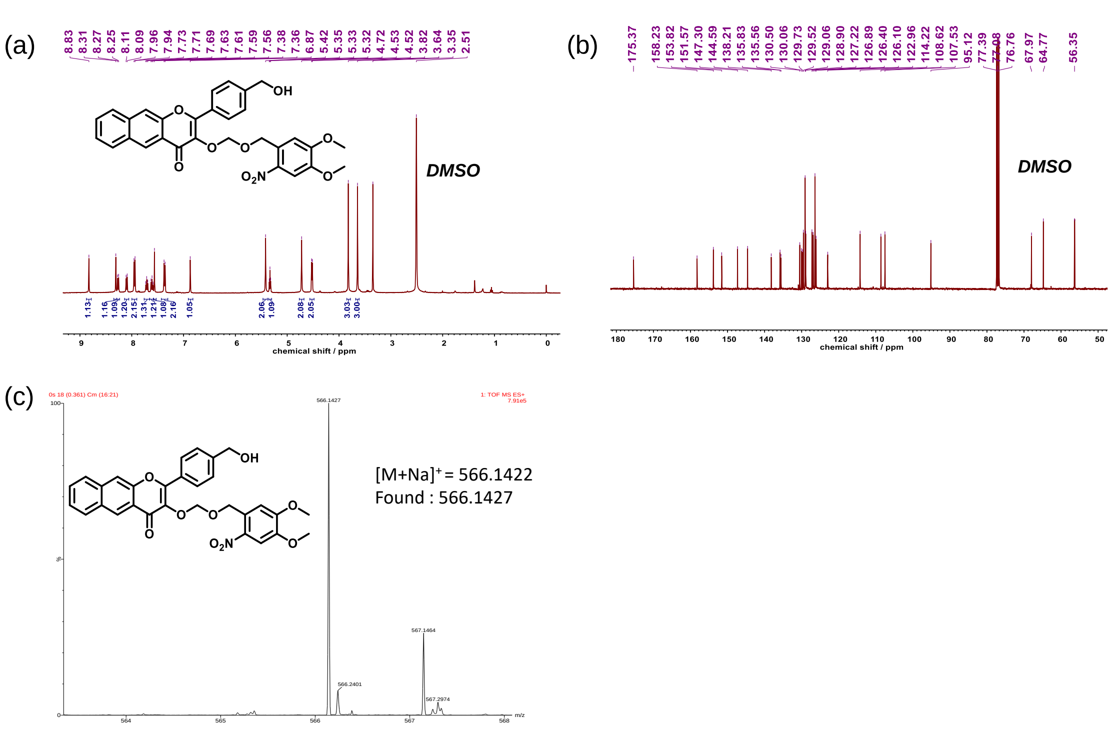

Figure S1 (a) ^{1}H NMR spectra recorded in DMSO-d_6 for compound A; (b) ^{13}C NMR spectra recorded in DMSO-d_6 for compound A; (c) ESI mass spectrum recorded for compound A in acetonitrile.

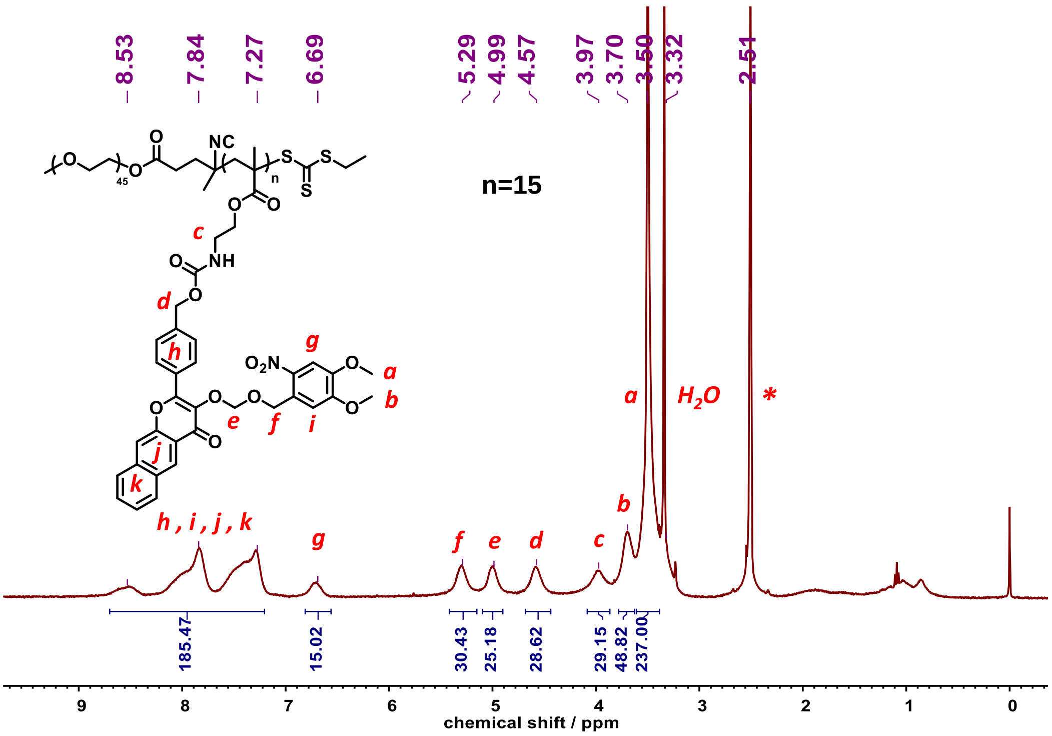

Figure S2 ^{1}H NMR spectra recorded in DMSO-d$_6$ for PEO$_{45}$-b-PFFM$_{15}$ (PCOFA).

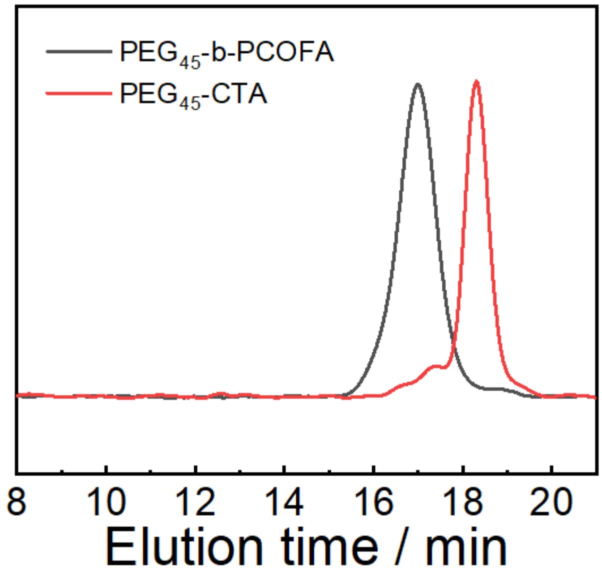

Figure S3 GPC Elution profiles of PEG$_{45}$-based macroinitiator and PCOFA.

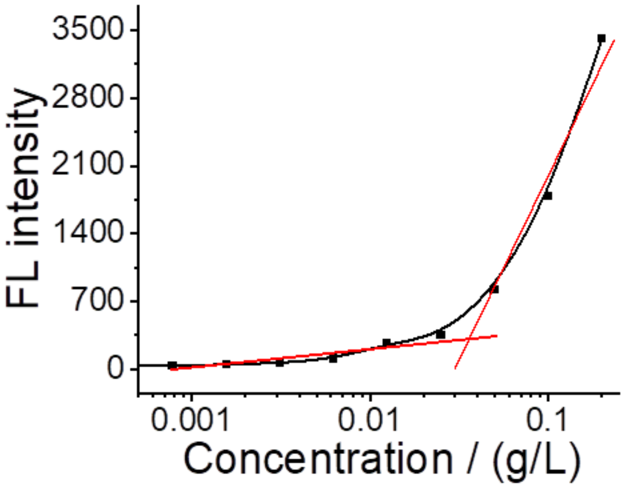

Figure S4 Concentration-dependent changes of surface tension of aqueous dispersions of PCOFA diblock copolymer.

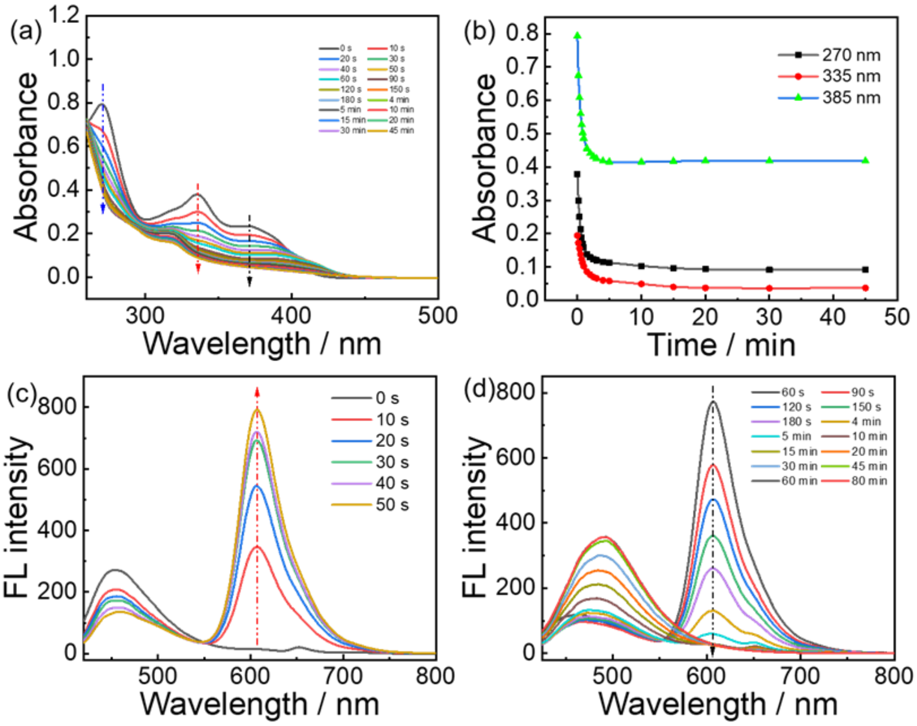

Figure S5 UV absorption spectra (a) and trend diagram of main characteristic absorption peaks with illumination time (b) of Compound B (80 μM, DMSO/H_2O, v/v=8/2) irradiated by 410 nm LED lamp (28 mW/cm^2). The fluorescence emission spectrum of B from 0-50 s (c) and 60s-90s (d) (λ_{ex} = 405 nm; slit widths: $E_{x.}$ = 5.0 nm, $E_{m.}$ = 5.0 nm).

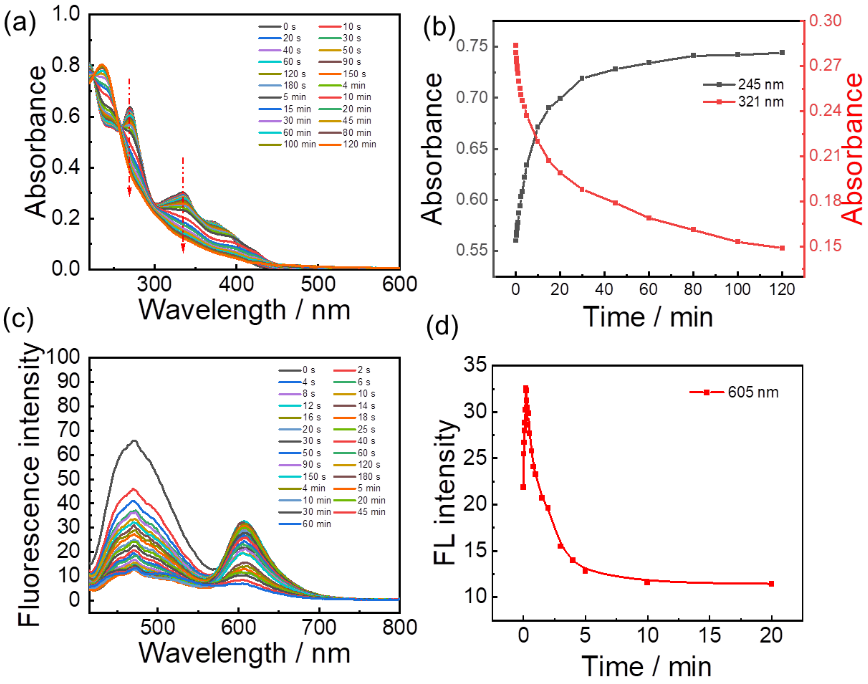

Figure S6 UV absorption spectra (a) and trend profile of main characteristic absorption peaks with illumination time (b) of PCOFA micelle solution (0.1 g/L) irradiated by 410 nm LED lamp (28 mW/cm^2). The fluorescence emission spectrum (c) and trend profile of main characteristic absorption peaks with illumination time (d) of PCOFA micelle solution (0.1 g/L) (λ_{ex} = 405 nm; slit widths: $E_{x.}$ = 5.0 nm, $E_{m.}$ = 5.0 nm).

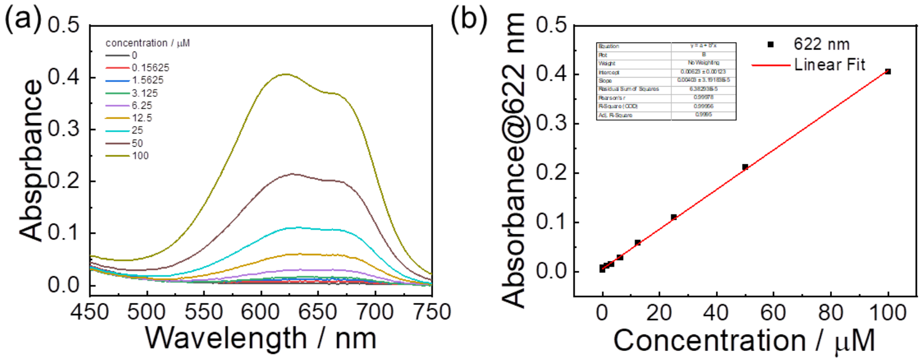

Figure S7 UV absorption spectra of (a) different concentrations of FA in water and (b) linear fitting standard curve at 622 nm.

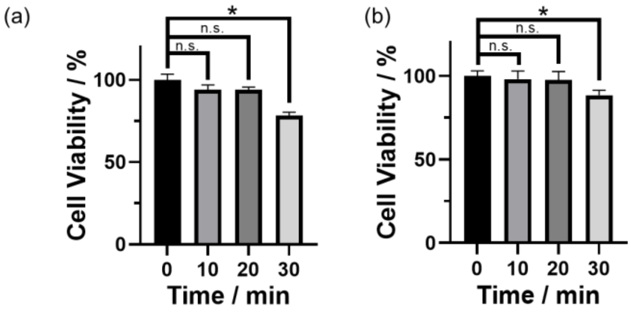

Figure S8 Cell viability of (a) HeLa and (b) L929 under 410 nm irradition（28 mW/cm^2）for varying duration after 24 hours incubation. Data are presented as mean± s.d. (n =3); n.s., not significant; p values were calculated in comparison with the non-irradiated groups. *$p < 0.05$; ** $p < 0.01$.

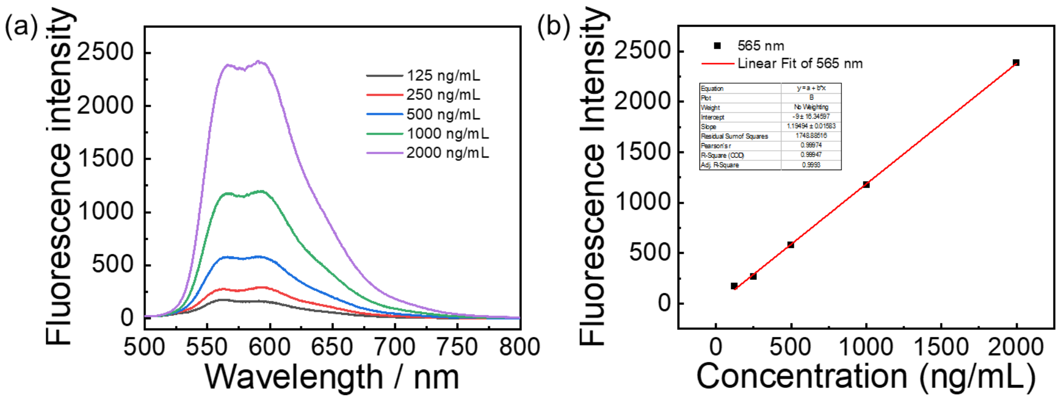

Figure S9 (a) Fluorescent spectra of DOX at different concentrations in DOMSO（λ_{ex}= 480 nm；slit widths：E_x. = 10 nm，E_m. = 10 nm)（b）Fitting curve of DOX concentration and fluorescence intensity at 565 nm.

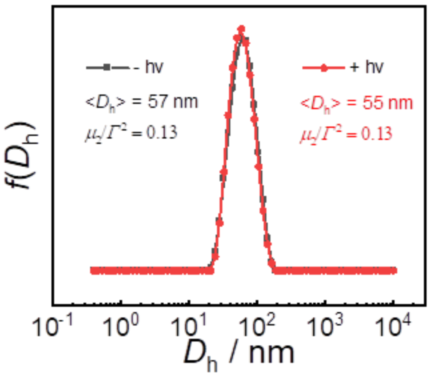

Figure S10 Number-average hydrodynamic distributions of PCOFA micelles without and with light irradiation. In all cases, the irradiation wavelength was 410 nm and the intensity was 28 mW/cm^2.
